# ﻿Investigation on the true identity of *Entomobryanigriventris* Stach, 1929 (Collembola, Entomobryidae) with the description of a new species

**DOI:** 10.3897/zookeys.1185.112279

**Published:** 2023-12-05

**Authors:** Daniel Winkler, Jakub Sternalski, Gábor Ónodi, Nóra Szigeti, Norbert Florián, László Dányi

**Affiliations:** 1 University of Sopron, Faculty of Forestry, Institute of Wildlife Management and Vertebrate Zoology, Bajcsy-Zs. str. 4, H–9400 Sopron, Hungary University of Sopron Sopron Hungary; 2 Institute of Systematics and Evolution of Animals, Polish Academy of Sciences, Sławkowska 17, Pl–31 - 016 Kraków, Poland Institute of Systematics and Evolution of Animals, Polish Academy of Sciences Kraków Poland; 3 National Laboratory for Water Science and Water Security, HUN-REN Balaton Limnological Research Institute, Tihany, Hungary, Klebelsberg Kuno u. 3, H–8237 Tihany, Hungary HUN-REN Balaton Limnological Research Institute Tihany Hungary; 4 Hungarian Research Institute of Organic Agriculture (ÖMKi), Villányi u. 29–43, H–1118 Budapest, Hungary Hungarian Research Institute of Organic Agriculture Budapest Hungary; 5 Institute for Soil Sciences, HUN-REN Centre for Agricultural Research, Herman Ottó út 15, H–1022 Budapest, Hungary Institute for Soil Sciences Budapest Hungary

**Keywords:** Central Europe, chaetotaxy, colour form, dark ventral colouration, dorsal macrochaetae formula, Entomobryni, taxonomy

## Abstract

The present paper gives a detailed and illustrated redescription of *Entomobryanigriventris* Stach, 1929, and the description of a new species collected from open sand steppe habitat in Hungary. Based on the colour pattern, *E.arenaria* Winkler, Flórián & Dányi, **sp. nov.** is close to *E.violaceolineata* Stach, 1963 but differs from it by the morphology of the labral papillae and the dorsal macrochaetotaxy of the head, Th II, and Abd II–IV. The new species is also characterised by dark ventral body colouration in adult specimens. In this regard, an overview of European *Entomobrya* species in which the dark ventral side may occur is also provided.

## ﻿Introduction

*Entomobrya* Rondani, 1861 is a widespread genus currently represented by 340 described species worldwide (Bellinger et al. 1996–2023). The checklist of the Hungarian Collembola fauna reports 20 species of this genus (Dányi and Traser 2008), including the historic species *E.nigriventris* Stach, 1929, originally described by Jan Stach from a single specimen collected by pharmacist and entomologist Ferenc Pillich in Simontornya, Hungary. In his comprehensive work on Entomobryni, Stach (1963) redescribed the species based on the same single specimen. Nevertheless, both descriptions were limited to the colour pattern and a few additional characters but not including any chaetotaxic pattern. Since the pioneering works of Szeptycki (1972, 1979), the dorsal chaetotaxy of the head, thorax, and abdomen proved to be the most informative and useful characters in species descriptions in Entomobryidae. For the genus *Entomobrya*, Jordana and Baquero (2005) proposed a set of characters that have become a standard for species identification and delimitation (e.g., Jordana et al. 2011; Jordana 2012; Jordana and Greenslade 2020; Baquero et al. 2021).

The key character of *E.nigriventris* is the almost entirely dark bluish black coloured ventral side of the body. Nevertheless, *E.nigriventris* is not the only European species of the genus having this feature, which can even lead to misidentification. Most likely based solely on the dark-coloured ventral side, *E.nigriventris* has been frequently reported from Hungary, collected from open sand steppes in Danube-Tisza Mid-Region (e.g., Hornung 1986; Loksa 1987; Traser 2002). Nevertheless, based on a re-examination and revision of these materials, we conclude that this species has been misreported as *E.nigriventris* while representing a different, new species we describe in this paper.

The “true” *E.nigriventris* has never been recollected from its actual type locality, which we managed to identify with great certainty (Szita et al. 2014), characterised by loess steppe vegetation. The present paper provides a detailed and illustrated redescription of *E.nigriventris* based on specimens newly collected from the type locality in Hungary. In order to minimise the possibility of further misidentification, we also provide an overview of other European *Entomobrya* species in which the dark ventral side may occur.

## ﻿Materials and methods

Since the single type specimen of *E.nigriventris* preserved at ISEA PAS is reportedly in poor condition, headless, and with no visible chaetotaxy (Jordana 2012), topotypic specimens were collected from the type locality (Simontornya, Hungary). Sampling of the *Entomobrya* material was carried out by using a D-Vac sampler. Specimens were extracted from the collected samples within three days using a Berlese apparatus without light or heating devices.

Specimens were cleared using Nesbitt fluid and then mounted on permanent slides in Hoyer’s medium. The slides were examined under a Leica DM2500 LED microscope with conventional bright light and phase contrast.

### ﻿Terminology

For the taxonomic description, the following nomenclatures were used: macrochaetotaxy of thoracic and abdominal segments follows Szeptycki (1979), while head chaetotaxy follows Soto-Adames (2008) and Jordana (2012). Interocular chaetae nomenclature follows Mari Mutt (1986). The system established by Gisin (1964) was followed for labial chaetotaxy. For labial palp, the notation of Fjellberg (1999) was used. Tergal specialised chaetae (S-chaetae) pattern follows Zhang and Deharveng (2015).

**Figure 1.**
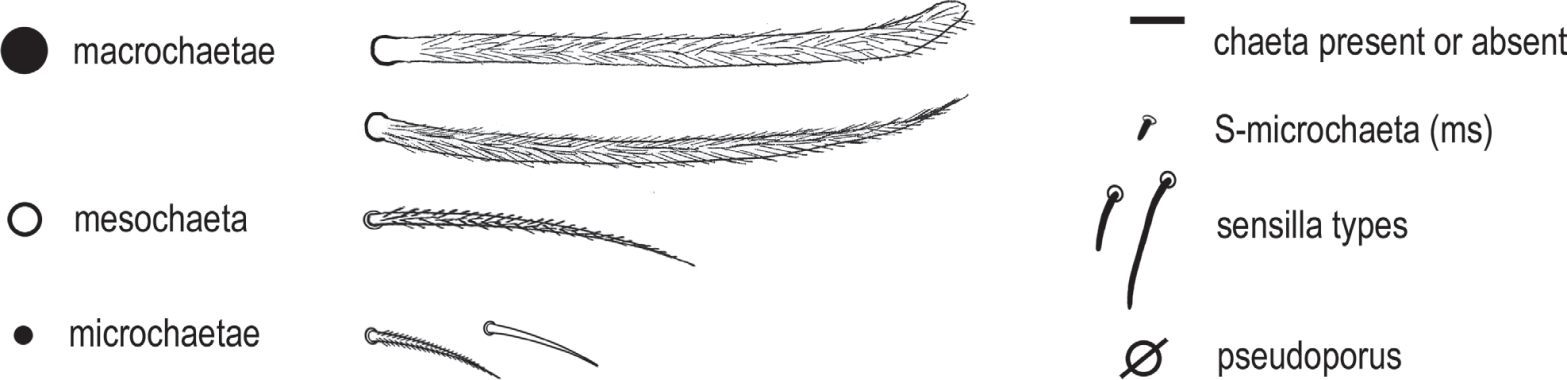
*Entomobrya*, symbols used for chaeta types and pseudopore.

Chaeta types and symbols used in detailed chaetotaxy schemes are shown in Fig. 1.

### ﻿Abbreviations used in the text and figures

**Abd** abdominal tergite

**acc.p** accessorial p-sensilla

**Ant** antennal segment

**a.s.l.** above sea level

**HNHM** Hungarian Natural History Museum, Budapest, Hungary

**ISEA PAS** Institute of Systematics and Evolution of Animals of the Polish Academy of Sciences, Krakow, Poland

**SOE** University of Sopron, Faculty of Forestry, Sopron, Hungary

**Mac** macrochaeta

**mes** mesochaeta

**mic** microchaeta

**psp** pseudopore

**Th** thoracic tergite

## ﻿Taxonomy

**Class Collembola Lubbock, 1870**

**Order Entomobryomorpha Börner, 1913, sensu Soto-Adames et al. 2008**

**Superfamily Entomobryoidea Womersley, 1934 sensu Zhang et al. 2019**

**Family Entomobryidae Tömösváry, 1882**

**Subfamilia Entomobryinae Schäffer, 1896 sensu Zhang and Deharveng 2015**

**Genus *Entomobrya* Rondani, 1861**

### ﻿ Entomobrya arenaria

Taxon classificationAnimaliaEntomobryomorphaEntomobryidae

Winkler, Flórián & Dányi sp. nov.

FCFBA1C0-4E11-5B47-8C86-BE5FCFDACFFA

https://zoobank.org/BFBAE815-66D4-4689-9BB1-7E2E7635F264

Figs 2 , 3 , 4

#### Type material.

***Holotype***: ♂ on slide (slide number HNHM-collpr-911), Hungary, Bács-Kiskun county, Fülöpháza, 106 m a.s.l., 46°52'16"N, 19°25'14"E, D-vac sample, 12 Jun. 2020, leg. D. Winkler and G. Ónodi. ***Paratypes***: ♀ on slide (slide number HNHM-collpr-912) and six ♂♂ on slide (slide numbers HNHM-collpr-913 to HNHM-collpr-914; WD–coll–141 to WD–coll–144, respectively); same data as holotype. The holotype and three paratypes are deposited at HNHM. Four paratypes are preserved at SOE in the first author’s collection.

#### Diagnosis.

Body orange-yellow, with thin dark dorsal centreline, dark transverse stripes anteriorly on Th II–Abd IV and Abd VI, and dorsomedial rectangular patch posteriorly on Abd IV. Ventral body entirely dark in adults. Ant IV with trilobed apical bulb. Labral papillae with spine-like projection. Lateral process on labial papilla E not reaching apex of papilla. Claw with four inner teeth. Paired lateral teeth and dorsal tooth intermediate. The exact identification of the species can be made by using the abbreviated macrochaetotaxy formula (sensu Jordana and Baquero 2005) of the head (H1–5 areas), Th II (T1–2 areas), Abd II (A1–2 areas), Abd III (A3–5 areas), and Abd IV (A6–10 areas) as: 5(6)-1(2)-0-3-2/4-5(6)/2-5(6)/0-2-2/0-4(6)-1_0_(0)4-1_0_(0)3(5)-2.

#### Description.

**Figure 2.**
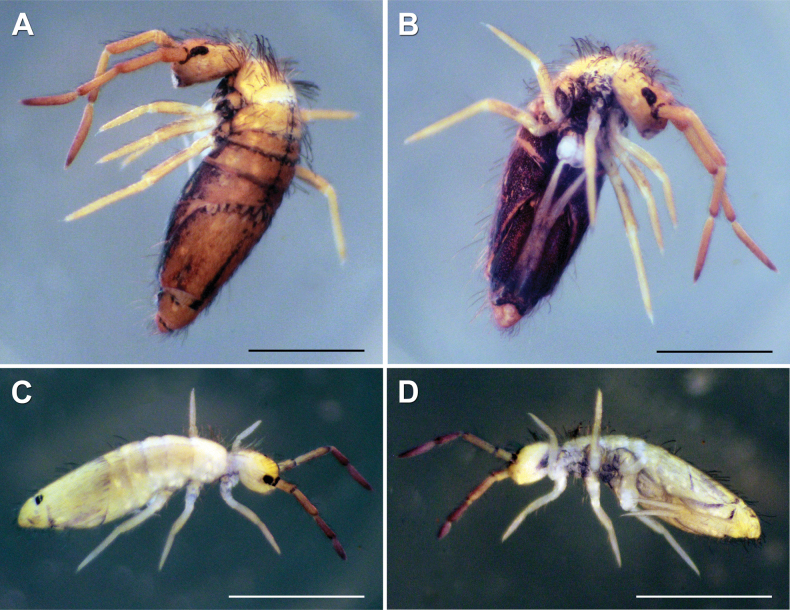
*Entomobryaarenaria* sp. nov. Habitus **A** adult specimen, dorsolateral view **B** same adult specimen ventral view **C** subadult specimen, dorsolateral view **D** same subadult specimen, dorsoventral view. Scale bars: 1 mm.

***Habitus*.** Adult body length (excluding antennae) 2.79–3.41 mm (*n* = 8), holotype 3.41 mm. Adult body ground colour orange-yellow (Fig. 2A, B), juveniles and subadults pale yellow (Fig. 2C, D). Pattern with a thin dark longitudinal line along dorsal centreline of Th II–Abd IV, widened towards end of Abd IV segment (in juvenile specimens, thin middle dorsal line purple and from Th II to Abd III only). Dark narrow, continuous, or occasionally interrupted transverse stripes on anterior margins of Th II–Abd IV and Abd VI. In juveniles, transverse stripes either very thin or missing. Posteriorly on Abd IV, a dorsomedial dark rectangular patch always present, both in adult and juvenile specimens. Antenna base black, black spot between bases of antennae. Dark violet pigment on antennae with increasing intensity from base to apex of segments. Lateral parts of abdominal segments and ventral body entirely dark in adult specimens. Ventral side in juveniles with no dark pigmentation, appearing first between legs in later developmental stages (Fig. 2D). In adults, dark pigmentation also on coxae and manubrial base.

**Figure 3.**
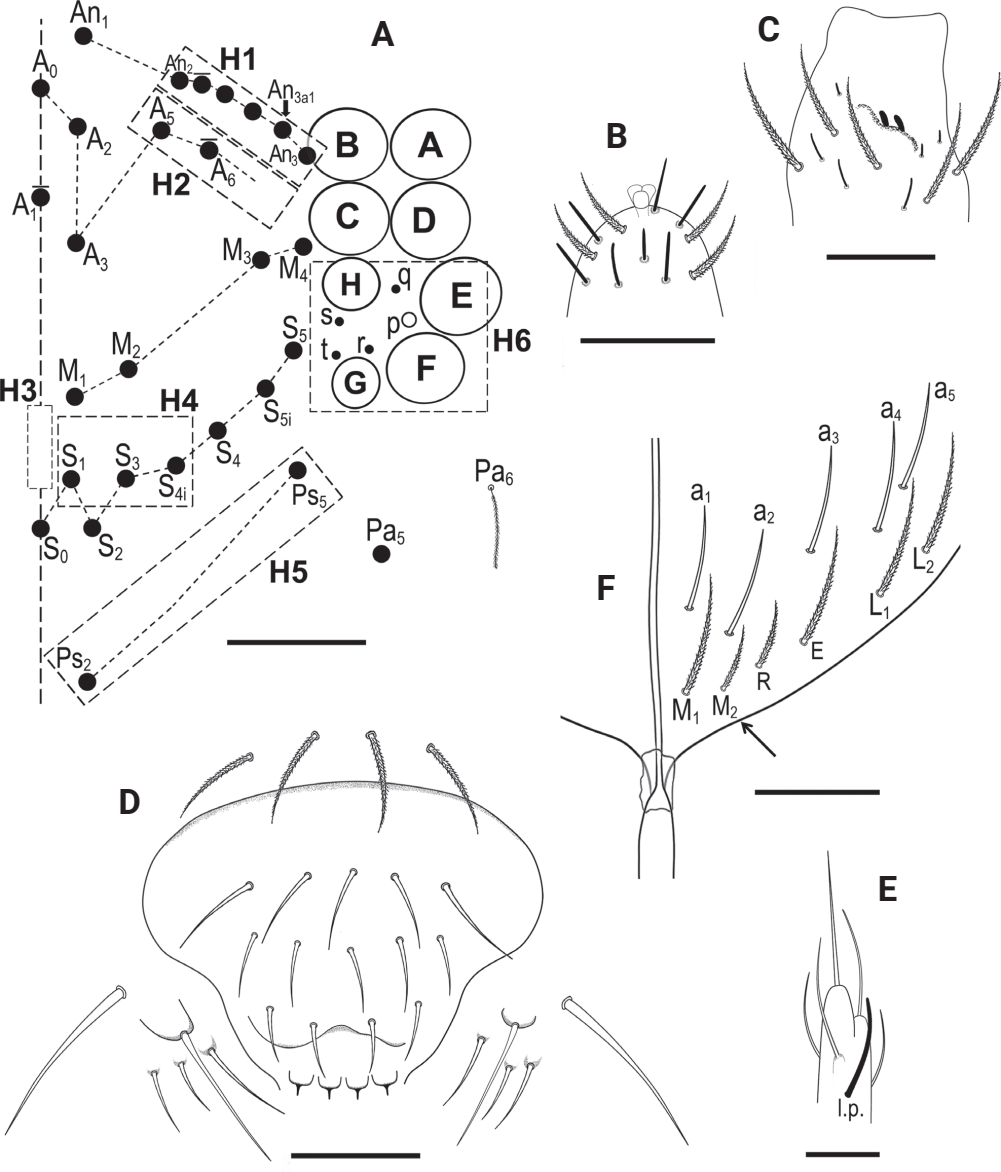
*Entomobryaarenaria* sp. nov. **A** head chaetotaxy **B** apex of Ant IV **C**Ant III sensillar organ **D** labrum with labral papillae, maxillary palp and sublobal plate **E** labial papilla E with lateral process (l.p.) **F** labial triangle, arrow indicates M_2_ chaeta present or absent. Abbreviations: Ant = antennal segment. Scale bars: 0.05 mm (**A**); 0.02 mm (**B, D**); 0.03 mm (**C, F**); 0.01 mm (**E**).

***Head*.** 8+8 eyes, GH smaller than EF (Fig. 3A). Interocular chaetotaxy with five chaetae (s, t, p, q, r). Antennae length 1.41–1.73 mm (*n* = 8), holotype 1.73 mm. Antennal length to head diagonal length ratio 2.50–2.77 (*n* = 8), holotype 2.50. Relation of antennal joints I–IV as 1: 1.8–2.3: 1.4–2.0: 1.8–2.3 (*n* = 7). Ant IV with trilobed apical bulb (Fig. 3B). Ant III sensillary organ composed of two sensory rods partially behind a cuticular fold, guarded by three short sensilla (Fig. 3C). Arrangement of chaetae on labrum 4/554, prelabral chaetae ciliated, posterior, median and anterior labral chaetae smooth (Fig. 3D). Labrum with four rounded labral papillae with strong, armed spine-like projection (Fig. 3D). Outer maxillary palp with two smooth chaetae and three smooth sublobal chaetae. Lateral process (sensu Fjellberg 1999) on labial papilla E not reaching apex of papilla (Fig. 3E). Labium chaetotaxy formed by five smooth “a” chaetae and, in the basal row, by ciliated chaetae M_1_, M_2_, R, E, L_1_ and L_2_ (Fig. 3F); M_2_ thinner and shorter than M_1_, R reduced. Chaeta M_2_ present in four of eight type specimens, bilaterally present in two specimens, and absent in two specimens.

**Figure 4.**
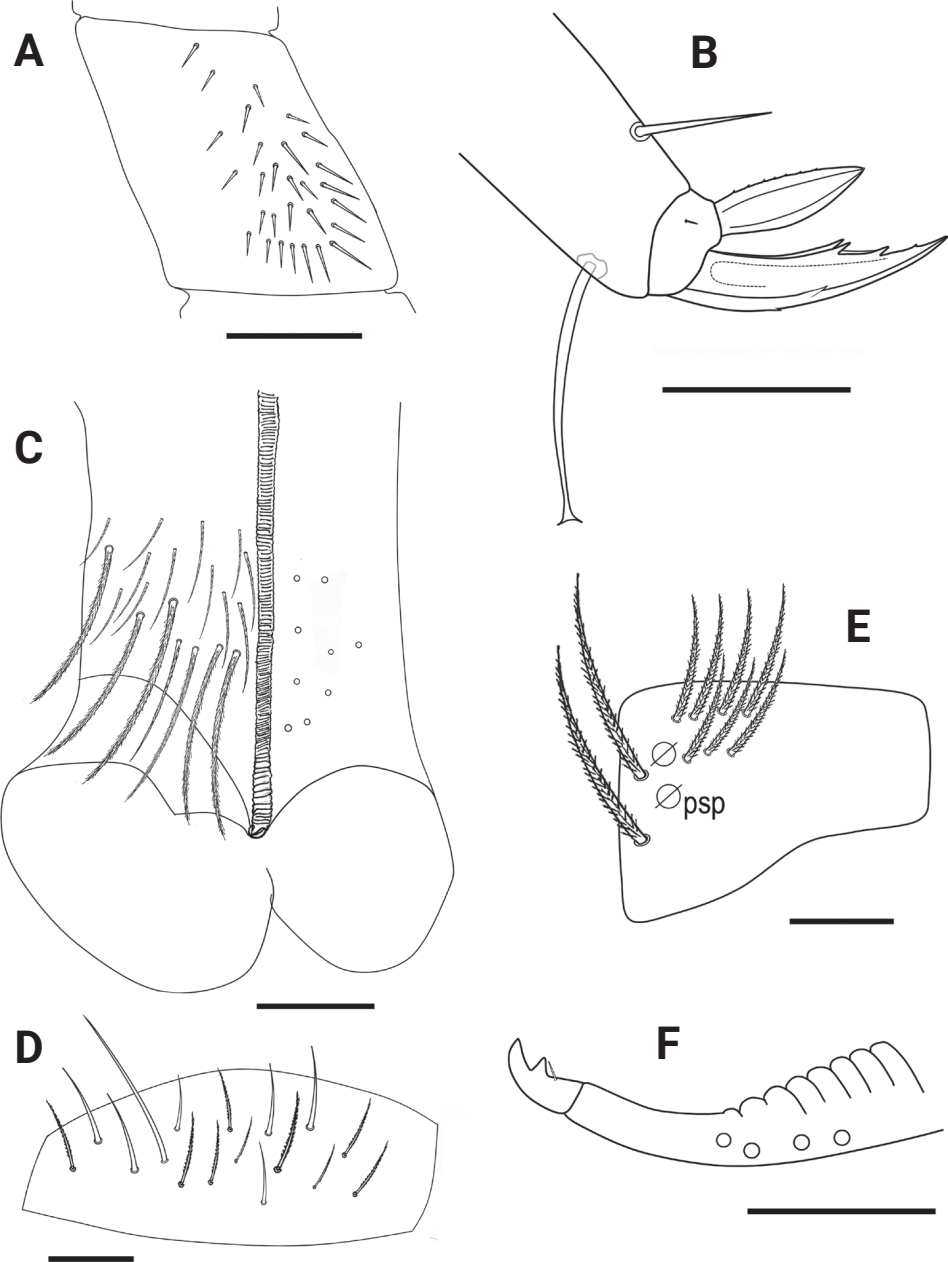
*Entomobryaarenaria* sp. nov. **A** trochanteral organ **B** unguis and unguiculus of leg III **C** ventral tube anterior view (right side) and posterior view (left side), circles indicate ciliated chaetae **D** ventral tube lateral flap **E** manubrial plate **F** mucro. Scale bars: 0.05 mm (**A, C**); 0.03 mm (**B, D, E–F**).

***Body*.** Ratios of Abd IV/III length 3.57–4.47 (*n* = 8), holotype 4.23. No differentiated chaetae on tibiotarsus III, with exception of smooth terminal chaeta opposite to tenent hair. Trochanteral organ with up to 29 spine-like chaetae (Fig. 4A). Unguis and unguiculus of claw III as in Fig. 4B. Unguis inner side with sub-equal paired basal teeth at 54% from inner edge, and with two more unpaired teeth at 71% and 86% from inner edge, respectively. Paired lateral teeth intermediate, at level slightly below the paired internal teeth. Unpaired dorsal tooth located approximately at 35–45% of distance from base. A small pretarsal chaeta present on both anterior and posterior surfaces. Unguiculus lanceolate, outer lamella serrated. Tibiotarsal tenent hair clavate, as long as claw. Ratio of smooth terminal chaeta / unguiculus around 1. Ventral tube with 19+19 ciliated chaetae on anterior side and 9+9 ciliated chaetae on posterior side (Fig. 4C); lateral flap with nine ciliated and seven smooth chaetae (Fig. 4D). Manubrial plate with eight or nine chaetae, including two larger inner chaetae and six or seven chaetae outer two psp (Fig. 4E). Length of not ringed terminal dens ~ 2× the length of mucro. Mucro with distal tooth larger than anteapical one; basal spine just reaching tip of anteapical tooth (Fig. 4F).

**Figure 5.**
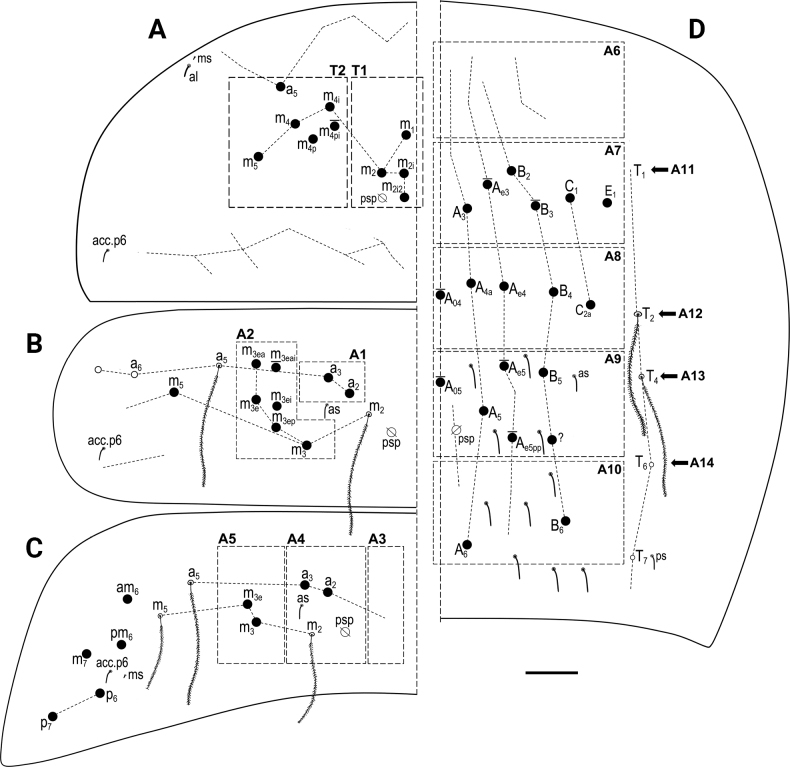
*Entomobryaarenaria* sp. nov. **A**Th II dorsal macrochaetotaxy **B**Abd II dorsal macrochaetotaxy **C**Abd III dorsal macrochaetotaxy **D**Abd IV dorsal macrochaetotaxy. Abbreviations: Abd = abdominal tergite; Th = thoracic tergite. Scale bar: 0.05 mm.

***Macrochaetotaxy*** (Figs 3A, 5A–D). Simplified Mac formula: 5(6)-1(2)-0-3-2/4-5(6)/2-5(6)/0-2-2/0-4(6)-1_0_(0)4-1_0_(0)3(5)-2. Head (Fig. 3A): H1 area with five Mac (An_2_, An_3a1_, An_3a2_, An_3_ and one additional Mac from the An series); H2 area regularly with one Mac (A_5_) and occasionally (and always bilaterally) also with A_6_ as Mac; H3 area without Mac; H4 area with three Mac (S_1_, S_3_ and S4_i_); H5 area with two Mac (Ps_2_ and Ps_5_). Mesothorax (Fig. 5A): area T1 with four Mac (m_1_, m_2_, m_2i_ and m_2i2_); T2 with 5–6 Mac (a_5_, m_4_, m_4i_, m_4p_ always present, m_4pi_ present or absent). Abdomen: Abd II (Fig. 5B) area A1 with two Mac (a_2_ and a_3_); area A2 with 5–6 Mac (m_3_, m_3e_, m_3ep_, m_3ei_ and m_3ea_ always present, m_3eai_ present or absent); Abd III (Fig. 5C) area A3 without Mac; area A4 with two Mac (a_2_ and a_3_), and area A5 with two Mac (m_3_ and m_3e_); Abd IV (Fig. 5D) area A6 without Mac; area A7 with 4–6 Mac (A_3_, B_2_, C_1_ and E_1_ always present; A_e3_ present in half of the studied specimens while B_3_ in a quarter); area A8 with unpaired central Mac A_04_ present or absent, and four Mac (A_4a_, A_e4_, B_4_ and C_2a_); area A9 with unpaired central Mac A_05_ present or absent, and 3–5 Mac (A_5_, B_5_ and one Mac of uncertain homology always present, A_e5_ and A_e5pp_ present or absent); and area A10 with two Mac (A_6_ and B_6_); sensillar formula from Th II to Abd V: 2,2/1,2,2,12,3; microsensillar formula from Th II to Abd III: 1,0/1,0,1.

#### Ecology and distribution.

**Figure 6.**
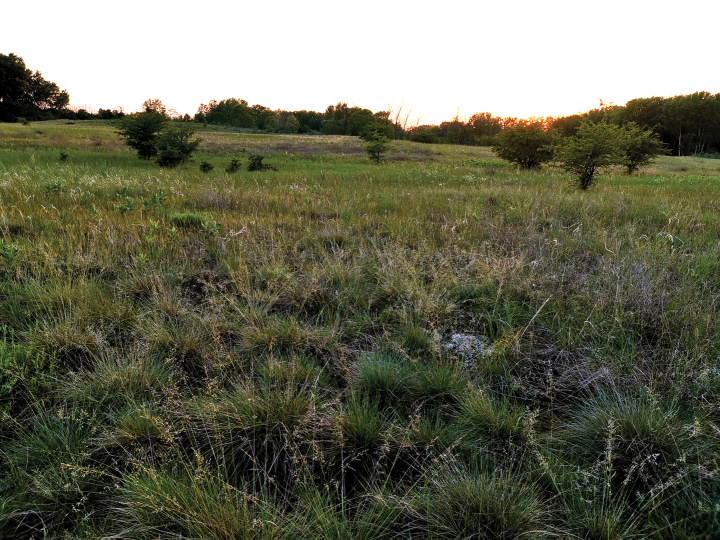
*Entomobryaarenaria* sp. nov. Type locality in Pannonic sand steppe grassland (Fülöpháza, Hungary).

The habitat of the type locality is extremely xerophilic. It belongs to the Pannonic sand steppes, where the vegetation is a partly opened grass dominated by *Festucavaginata* and *Stipaborysthenica* (Fig. 6).

#### Etymology.

The name of the new species refers to the habitat, Pannonic open sand steppes, where *E.arenaria* is one of the most dominant epigeic Collembola species.

#### Remarks.

Based on the colour pattern, *E.arenaria* sp. nov. is very close to *E.violaceolineata* Stach, 1963, with the difference that, in the case of the latter, neither the original description (Stach 1963) nor other descriptions (Jordana 2012) mention the presence of a dark ventral side, which is a key character of the new species. Stach (1963) only notes that, similarly to *E.schoetti* Stach, 1922, the dark-pigmented body side often appears also in individuals of *E.violaceolineata*. *Entomobryaarenaria* sp. nov. differs from *E.violaceolineata* by the morphology of the labral papillae (rounded with one strong-armed spine-like projection in the new species while truncate and bearing three short setulae in *E.violaceolineata*). There is a further difference regarding the apical bulb of the fourth antennal segment, trilobed in the new species, while simple (sensu Stach 1963) or bilobed (sensu Jordana 2012) in *E.violaceolineata*. The shape of unguiculus is also different, as its external edge is serrate in the new species while smooth in *E.violaceolineata*. The Abd IV/III ratio of *E.arenaria* sp. nov. is above 4, while Abd IV of *E.violaceolineata* is relatively short, resulting in a smaller (~3) Abd IV/III ratio. Based on specimens collected in Spain, Jordana (2012) was the first to provide the complete macrochaetotaxy for *E.violaceolineata*, compared to which marked differences in the number of macrochaetae can be observed in most areas, including head H1, H4, and H5; Th II T2; Abd II A2; Abd III A3 and A5; Abd IV A6, A7, and A8, respectively (Table 1). Regarding habitat characteristics, *E.arenaria* sp. nov. inhabits xerophilic open grasslands, while *E.violaceolineata* has been found under dead leaves and litter in parks, pine and riparian forest litter, and belts of meadows along river banks (Stach 1963; Jordana 2012; Buşmachiu et al. 2017).

**Table 1.** Set of diagnostic morphological characters of *Entomobrya* species most similar to *E.arenaria* sp. nov. in terms of Abd II–III macrochaetotaxy (*E.armeniensis*, *E.cheni*, *E.handschini*, *E.hirsutothorax*, *E.kuznetsovae*, *E.murreensis*, *E.nigriventris*, *E.pazaristei*, *E.strigata*, *E.taigicola*) and dorsal colour pattern (*E.violaceolineata*).

	*E.armeniensis*	*E.cheni*	*E.handschini*	*E.hirsutothorax*	*E.kuznetsovae*	*E.murreensis*	*E.nigriventris*	*E.pazaristei*	*E.strigata*	*E.taigicola*	*E.violaceolineata*	*E.arenaria* sp. nov.
**Ch1**	4*	3*	3(4)*	3(4)*	3*	4*	4(5)	2*	4*	3*	3*	5(6)
**Ch2**	1	1	1	1	1	2	1	2	2	3*	1	1(2)
**Ch3**	0	0	0	0	0	0	0	0	0	0	0	0
**Ch4**	3	3	3	3	3	3	3	3	3	3	1*	3
**Ch5**	2	2	2	2	2	3*	1*	3*	2(3)	2	3*	2
**Ch6**	3	3	3	3	3	3	3	3	1^1^ or 2-3^2^	na	2*	3
**Ch7**	2	2	2	2	2	2	2	2	2	2	2	2
**Ch8**	1*	na	3	1*	1*	2*	2-3	1*	2*	1*	1^1^ or 2^2^ *	3
**Ch9**	1*	na	2	1-2	2	1*	2	2	2	2	2	2
**Ch11**	2*	4	4	7(9)*	6*	7(8)*	4	8*	3*	6*	4	4(5)
**Ch12**	6	6	5(6)	7(11)*	6	3*	5	5	5	6	3*	5(6)
**Ch14**	4	4	4	4	4	4	3-4	4	4	4	4	4
**Ch15**	2	2	2	2	2	1*	2	1*	2	2	1*	2
**Ch17**	0*	0*	0*	0*	0*	0*	0*	0*	0*	0*	0*	1
**Ch18**	2	2	2	2	2	2	2	2	2	2	2	2
**Ch19**	7*	5(6-7)	5	4(5)	5	7*	5	5	5(7)	5	2*	5(6)
**Ch20**	0(1)	0	0	0	0	0	0	0	0	0	1*	0
**Ch21**	2	2	2	2	2	2	2	2	2	2	2	2
**Ch22**	2	2	2	2	2	2	2	2	2	2	1*	2
**Ch23**	0	0	0	0	3*	5*	0	9*	0	0	0	0
**Ch24**	0	0	0(1)	0	0	0	0	1*	0	0	0	0
**Ch25**	6	7(11)*	3(5)	5	4	3*	3(6)	6	3*	6	1*	4(6)
**Ch26**	0	0	0(1)	1	0	1	0(1)	1	0	0	0	0(1)
**Ch27**	3*	7(10)*	3(4)	4	3*	1*	4	4	3*	1*	3*	4
**Ch28**	0	0	0(1)	1	0	1	0	1	0	0	0	0(1)
**Ch29**	3	3(5)	3(5)	2(3)	4	4	3(4)	5	3	5	2*	3(5)
**Ch30**	2	8(10)*	2	3*	2	4*	2(3)	3*	2	3*	2	2
**Ch35**	2	2	1-2	2	1*	2	2	2	2	2	1*	2
**Ch36**	7*	3-5*	4-6*	5*	7*	na	5*	11*	4*	5*	4*	8-9
**Ch37**	2	2	2	2	2	na	2	2	2	2	2	2
**Ch38**	2*	2*	4	2*	2*	2*	4	2*	4	2*	4	4
**D**	9	7	3	8	9	14	3	11	7	9	16	

**Ch1** H1 area (head): number of Mc on series An_2_-An_3_; **Ch2** H2 area (head): number of Mc on series A_5_–A_7_; **Ch3** H3 area (head): chaeta S’_0_ absent (0) or present (1); **Ch4** H4 area (head): number of Mc on series S_1_-S_3_-S_4_; **Ch5** H5 area (head): number of Mc on series Ps_2_-Ps_3_-Ps_5_; **Ch6** labral papilla shape: simple and smooth (1); multispinose or with some projections (2); with a chaeta-like projection (3); **Ch7** Eyes G&H size: =E&F (1); <E&F (2); **Ch8** retractile apical antennal bulb: simple (1); bilobed (2); trilobed (3); **Ch9**Ant/Head ratio: > or = 3 (1); > or = 2 < 3 (2); < 2 (3); **Ch11** T1 area (Th II): number of Mc on series m_1_-m_2i2_; **Ch12** T2 area (Th II): number of Mc on series a_5_, m_4_–m_5_; **Ch14** number of unguis internal teeth; **Ch15** unguis dorsal tooth: basal (1); internal teeth level or not basal (2); **Ch17** external edge of unguiculus: smooth (0), serrate (1); **Ch18** A1 area (Abd II): number of on series Mc a_2_–a_3_; **Ch19** A2 area (Abd II): number of Mc on series m_3_; **Ch20** A3 area (Abd III): Mc a_1_ absent (0) or present (1); **Ch21** A4 area (Abd III): number of Mc above bothriotrichum m_2_; **Ch22** A5 area (Abd III): number of Mc on series m_3_-m_4_; **Ch23** A6 area (Abd IV): number of Mc on series A_1_-D_1_; **Ch24** A7 area (Abd IV): unpaired Mc A_03_ absent (0) or present (1); **Ch25** A7 area (Abd IV): number of Mc on series A_2_–E_1_; **Ch26** A8 area (Abd IV): unpaired Mc A_04_ absent (0) or present (1); **Ch27** A8 area (Abd IV): number of Mc on series A_4_-C_2a_; **Ch28** A9 area (Abd IV): unpaired Mc A_05_ absent (0) or present (1); **Ch29** A9 area (Abd IV): number of Mc on series A_5_–B_5_; **Ch30** A10 area (Abd IV): number of Mc on series A_6_–B_6_; **Ch35**Abd IV/III ratio: 2 < R < 4 (1); R > 4 (2); **Ch36** manubrial plate: number of chaetae; (11) if > 10; **Ch37** manubrial plate: number of pseudopore; **Ch38** mucro: sub-apical tooth: without (1); normal (2); larger than apical (3); smaller than apical (4); **D** Total number of differences between the new species and the other species. * Differences in the characters of the species with respect to *E.arenaria* sp. nov. ^1^ sensu Stach (1963), ^2^ sensu Jordana (2012); na – information not available.

Upon further investigation, ten species share the same or very similar macrochaetotaxy of Abd II–III (Table 1), namely *E.armeniensis* Jordana, Potapov & Baquero, 2011; *E.cheni* Baquero, Arbea & Jordana, 2010; *E.handschini* Stach, 1922; *E.hirsutothorax* Jordana & Baquero, 2021 (in Baquero et al. 2021); *E.kuznetsovae* Jordana, Potapov & Baquero, 2011; *E.murreensis* Yosii & Ashraf, 1965; *E.nigriventris*; *E.pazaristei* Denis, 1933; *E.strigata* Stach, 1963; *E.taigicola* Jordana, Potapov & Baquero, 2011; respectively. While the colour pattern of the new species is quite different from the abovementioned species, there are differences also in chaetotaxy and other characters (Table 1). *Entomobryaarmeniensis* is characterised by a slightly different formula for the head and has an additional Mac (S_4p_) absent in the new species, fewer Mac on the T1 area of Th II, A8–A9 area of Abd IV. *Entomobryacheni* bears significantly more Mac on Abd IV areas A7, A8, and A10, respectively, and fewer chaetae on the manubrial plate compared with *E.arenaria* sp. nov. The dorsal macrochaetotaxy scheme of *E.handschini* is very close to that of the new species, with the only difference involving the area H1 on the head with fewer Mac (3 or 4); in addition, the smaller number of chaetae on the manubrial plate can be mentioned when compared to the new species. In the case of *E.kuznetsovae*, the simple apical bulb (trilobed in the new species) and the presence of macrochaetae in the A6 area in Abd IV (without Mac in the new species) can be highlighted as differential characters. *Entomobryamurreensis* differs from *E.arenaria* sp. nov., most notably by the macrochaetae formula of Th II and by the presence of macrochaetae in the A6 area in Abd IV. The dorsal macrochaetotaxy scheme of *E.nigriventris* and *E.strigata* is very close to that of the new species. In *E.strigata*, differences include fewer Mac in the area H1 on the head, T1 area of Th II, and A7–A9 areas of Abd IV. *Entomobryanigriventris* has only one Mac in the area H5 of the head and, similarly to *E.strigata*, has a significantly smaller number of chaetae on the manubrial plate. The cave-dwelling species *E.pazaristei* differs from the new species by the different Mac formula for the head, the higher number of Mac in the area T1 in Th II, and the presence of macrochaetae in the A6 area of Abd IV. *Entomobryataigicola* mainly differs from the new species by the higher number of Mac (3) in the area H2 on the head, the fewer number (1) of Mac in the area A8 of Abd IV, and fewer chaetae on the manubrial plate.

### ﻿ Entomobrya nigriventris

Taxon classificationAnimaliaEntomobryomorphaEntomobryidae

Stach, 1929

6F4164B3-8B89-5EA0-B29D-5BC542B4442A

Figs 7 , 8 , 9

Entomobrya nigriventris Stach, 1929: 302; Bonet 1934: 168 (keyed, diagnosis); Gisin 1944: 77 (keyed); Gisin 1960: 222 (keyed, diagnosis); Stach 1963: 16, 40–41, pl 9 figs 4–6 (keyed, redescribed): Palissa 1964: 204 (keyed, diagnosis); Hornung 1986: 138; Loksa 1987: 79; Jordana 2012: (keyed, diagnosis); Traser et al. 2006; Dányi and Traser 2008: 33; Flórián et al. 2019: 9.
Entomobrya cf. nigriventris : Arbea and Jordana 1985: 61; Jordana et al. 1990: 57, 218.

#### Material examined.

Nine topotypic specimens from type locality in Hungary, Simontornya, com. Tolna, Barcsi Valley, hillside with loess steppe meadow, 120 m a.s.l., 46°45'59"N, 18°31'50"E, D-vac sample, 10 Aug. 2021 (leg. D. Winkler, N. Szigeti and G. Traser): three ♂♂ on slides (slide numbers as Nr. HNHM-collpr-915 to HNHM-collpr-916; and WD–coll–145); two ♀♀ on slide (slide numbers as Nr. HNHM-collpr-917; and WD–coll–146); three juveniles on slide (slide numbers as Nr. HNHM-collpr-918; WD–coll–147 to WD–coll–148), deposited at HNHM, and in the first author’s collection at SOE.

#### Redescription.

**Figure 7.**
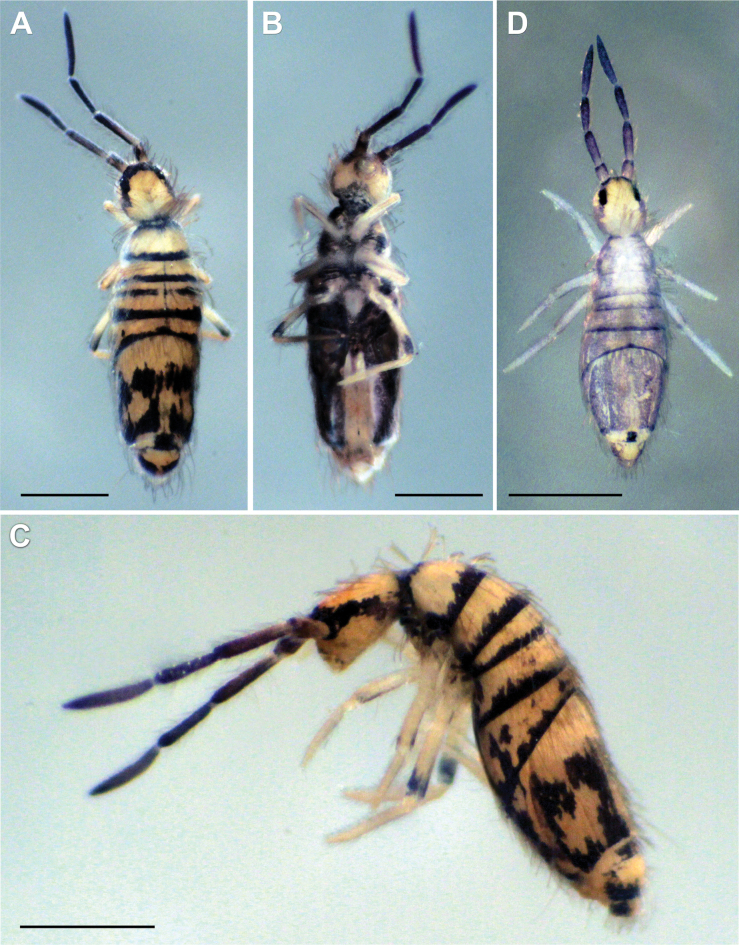
*Entomobryanigriventris.* Habitus **A** adult specimen, dorsal view **B** same adult specimen, ventral view **C** adult specimen, dorsolateral view **D** juvenile specimen, dorsal view. Scale bars: 0.5 mm.

***Habitus*.** Adult body length (excluding antennae) 1.20–2.49 mm (*n* = 7), holotype 1.20 mm (after Stach 1929). Body ground colour pale yellow (Fig. 7A–D). Adult colour pattern (Fig. 7A–C) characterised by a thin longitudinal stripe running along dorsal centreline, usually from Th II to Abd IV. Dark bluish black transverse stripes on anterior and posterior margins of Th II, and posterior margin of Th III to Abd III (broadest in Abd II). On each side of Abd IV, four irregular, broad, separate, or connected patches. Abd IV posteriorly with a dorsomedial rectangular patch. Abd V with two posterolateral patches. Head with broad dark band between antenna bases also connecting to eye patches and often continued longitudinally beyond them, in some cases reaching the lateral posterior part of the head. Dark violet pigment on antennae with increasing intensity from base to apex of segments. Juveniles (Fig. 7D) with similar pattern but without irregular patches on Abd IV. Ventral body entirely dark in most adult specimens, in one specimen only the area between legs pigmented (in juveniles, ventral side with no dark pigmentation). In adults, dark pigmentation also on coxae and manubrium.

**Figure 8.**
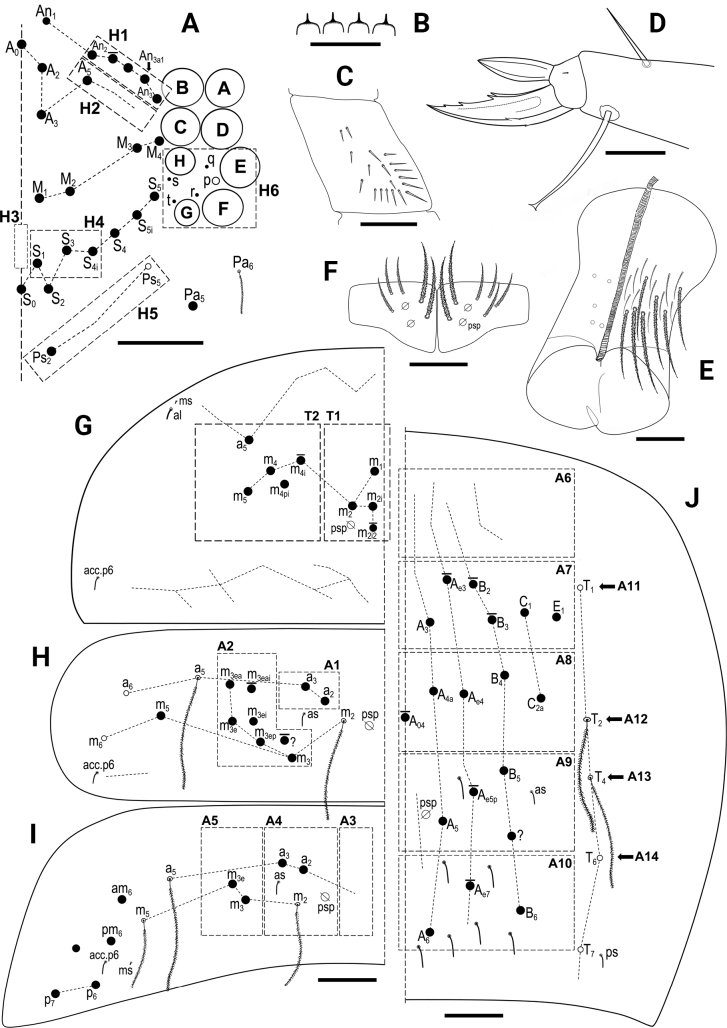
*Entomobryanigriventris***A** head chaetotaxy **B** labral papillae **C** trochanteral organ **D** unguis and unguiculus of leg III **E** Ventral tube anterior view (left side) and posterior view (right side), circles-ciliated chaetae **F** Manubrial plate **G**Th II dorsal macrochaetotaxy **H**Abd II dorsal macrochaetotaxy **I**Abd III dorsal macrochaetotaxy **J**Abd IV dorsal macrochaetotaxy. Abbreviations: Abd = abdominal tergite; Th = thoracic tergite. Scale bars: 0.03 mm (**A, D–F**); 0.02 mm (**B**); 0.05 mm (**C, G–J**).

***Head*.** 8+8 eyes, GH smaller than EF (Fig. 8A). Interocular chaetotaxy with five chaetae (s, t, p, q, r). Antennae length 0.75–1.32 mm (*n* = 4). Antennal length to head diagonal length ratio 2.17–2.69 (*n* = 6). Relation of antennal joints I–IV as 1: 1.64–2:22: 1.57–2.11: 2.25–3.00 (*n* = 6). Ant IV with bi- or trilobed apical vesicle. Ant III sensillary organ composed of two sensory rods partially behind a cuticular fold, guarded by three short sensilla. Arrangement of chaetae on labrum 4/554, prelabral chaetae ciliated, posterior, median and anterior labral chaetae smooth. Labrum with four rounded labral papillae with spine-like projection (Fig. 8B). Outer maxillary palp with two smooth chaetae and three smooth sublobal chaetae. Lateral process on labial papilla E not reaching apex of papilla. Labium chaetotaxy formed by five smooth “a” chaetae and, in basal row, by ciliated chaetae M_1_, R, E, L_1_, and L_2_ with R reduced (ratio of R/M_1_~0.5).

***Body*.** Ratio of Abd IV/III length 4.00–5.89 (*n* = 6). No differentiated chaetae on tibiotarsus III, with exception of the smooth terminal chaeta opposite to tenent hair. Trochanteral organ with up to 19 spine-like chaetae (Fig. 8C). Unguis and unguiculus of claw III as in Fig. 8D. Unguis inner side with sub-equal paired basal teeth at 50% from the inner edge, and with two more unpaired teeth at 72% and 87% from inner edge, respectively (holotype with three inner teeth on claw III, most distal unpaired one absent). Paired lateral teeth intermediate, at level slightly below the paired internal teeth. Unpaired dorsal tooth hardly observable, located approximately at 40% of distance from base. A small pretarsal chaeta present on both anterior and posterior surfaces. Unguiculus lanceolate, outer lamella smooth. Tibiotarsal tenent hair clavate, as long as claw. Ratio of smooth terminal chaeta / unguiculus around 1. Ventral tube with 19+19 ciliate chaetae of various size and 5+5 ciliated chaetae on posterior side (Fig. 8E); lateral flap with nine ciliated and seven smooth chaetae. Manubrial plate with five or six chaetae (Fig. 8F). Length of not ringed terminal dens ~ 2× the length of mucro. Mucro with subapical tooth somewhat smaller than apical one; basal spine just reaching tip of subapical tooth.

***Macrochaetotaxy*** (Fig. 8A, G–J). Simplified Mac formula: 4(5)-1-0-3-1/3(4)-4(5)/2-5(7)/0-2-2/0-3(6)-1_0_(0)4-3(4)-2(3).

Head (Fig. 8A): H1 area with four or five Mac (An_2_, An_3a1_, An_3a2_, and An_3_ always present, one additional Mac from the An series present or absent); H2 area with one Mac (A_5_); H3 area without Mac; H4 area with three Mac (S_1_, S_3_, S_4i_); H5 area with one Mac (Ps_2_), Ps_5_ present as mes. Mesothorax (Fig. 8G): area T1 with three or four Mac (m_1_, m_2_, m_2i_ always present, m_2i2_ present or absent); T2 with four or five Mac (a_5_, m_4_, m_4p_ always present, m_4i_ present or absent). Abdomen: Abd II (Fig. 8H) area A1 with two Mac (a_2_ and a_3_); area A2 with 5–7 Mac (m_3_, m_3e_, m_3ep_, m_3ei_, m_3ea_ always present, m_3eai_ present or absent; bilaterally an additional Mac present in one specimen); Abd III (Fig. 8I) area A3 without Mac; area A4 with two Mac (a_2_ and a_3_), and area A5 with two Mac (m_3_ and m_3e_); Abd IV (Fig. 8J) area A6 without Mac; area A7 with 3–6 Mac (A_3_, C_1_, E_1_ always present; A_e3_, B_2_, B_3_ present or absent); area A8 with unpaired central Mac A_04_ present or absent, and with four Mac (A_4a_, A_e4_, B_4_, C_2a_); area A9 with 3–4 Mac (A_5_, B_5_, and one Mac of uncertain homology always present, A_e5p_ present or absent); and area A10 with two or three Mac (A_6_ and B_6_ always present, Ae7 present or absent); sensillar formula from Th II to Abd V: 2,2/1,2,2,9,3; microsensillar formula from Th II. to Abd III: 1,0/1,0,1.

#### Ecology and distribution.

**Figure 9.**
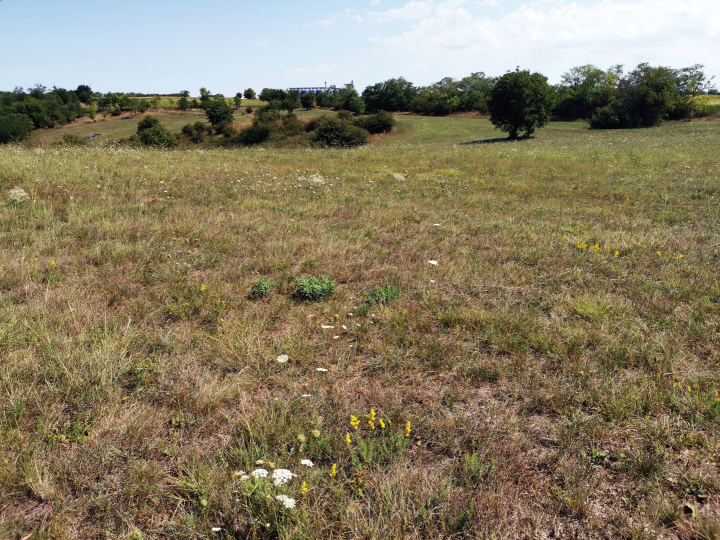
*Entomobryanigriventris.* Type locality (Simontornya, Hungary).

The type locality near the settlement Simontornya is situated on the loess ridges of the southern Transdanubian region of Hungary. According to historical maps, the area was pasture and mowed meadow centuries ago. Nowadays, the effects of intensive grazing can be observed on the grass vegetation, which consists of common species like *Bothriochloaischaemum*, *Galiumverum*, *Salviapratensis* (Fig. 9). Based on the habitat and climatic characteristics, the species can be considered xerophilic. Notably, we also detected some of the co-existent species Ferenc Pillich collected together with *E.nigriventris* and sent to Jan Stach for determination. These include species Stach described together with *E.nigriventris* in the same paper (Stach 1929): *Orchesellahungarica* Stach, 1929 and *Pseudosirapillichi* Stach, 1929, recently synonymised with *Seirapallidipes* Reuter, 1895 (Winkler and Dányi 2017), as well as other *Entomobrya* species such as *E.handschini* Stach, 1922 and *E.quinquelineata* Börner, 1901.

Until now, *E.nigriventris* has been known only from its type locality in Hungary (Simontornya). Although the species has been frequently reported from open sand steppes in Central Hungary (Hornung 1986; Loksa 1987; Traser 2002; Traser and Horváth-Szováti 2006; Flórián et al. 2019), these observations covered another species described in this article as *E.arenaria* sp. nov.. Traser et al. (2006) reported the species from West Hungary in a moss habitat, but re-examining their collected material proved this observation erroneous. In Spain, Arbea and Jordana (1985) detected a species (E.cf.nigriventris) that shows similarities based on the colour pattern. On the other hand, considering its habitat (beech forest, with moss cover in the more open parts), it is likely to represent another species.

#### Remarks.

*Entomobryanigriventris* was described based on a single specimen (Stach 1929), which entails the problem that the natural variability of the diagnostic characters cannot be determined. In addition to the new and essential information on the chaetotaxy and its variations, the examination of the newly collected specimens also allowed us to describe the size range and the colour pattern from the juvenile to the adult stage. Some of the already known characters (colour pattern, morphology of labral papillae, lateral process) are in accordance with the original description (Stach 1929) and later redescriptions (Stach 1963; Jordana 2012) based on the holotype. Morphology of Ant IV apical bulb can be bi- or trilobed (we found both variations in the newly collected specimens); in the holotype, it is trilobed (Stach 1963). This moderate intraspecific variation has already been reported in other *Entomobrya* species (Katz et al. 2015). However, there is a difference regarding the morphology of the claw III that must be mentioned. In the case of the holotype, the number of inner teeth on the claw is three, with only one (medial) unpaired tooth, while the apical unpaired tooth is reported to be absent (Stach 1963; Jordana 2012). In the freshly collected specimens from the type locality, an additional minute apical tooth was always present. This variability, although not common, may occur in *Entomobrya* (Jordana and Baquero 2006) and other derived genera of Entomobryomorpha, such as *Lepidocyrtus* (e.g., Mateos 2008; Winkler 2017), *Pseudosinella* (Winkler and Mateos 2018), and *Heteromurus* (Yoosefi Lafooraki and Shayanmehr 2014). It is also worth noting that Stach (1963) reported *E.quinquelineata* as a species with three inner teeth, while the original description (Börner 1901) and its redescriptions (Baquero and Jordana 2008; Jordana 2012) do not mention the absence of the apical unpaired tooth.

Apart from the dark ventral side and the presence of the longitudinal thin stripe running along the dorsal centreline, the colour pattern of *E.nigriventris* is very close to *E.strigata* Stach, 1963, originally described from Poland (Stach 1963). It can be, however, distinguished by the morphology of labral papillae (with spine-like projection in *E.nigriventris* and with plain surface in *E.strigata* sensu Stach (1963)). The holotype of *E.strigata* is reported to be lost; therefore, to describe the dorsal macrochaetotaxy and additional characters missing from the original description, specimens collected in Armenia were used by Jordana (2012). This allowed us to observe further differences between the two species in the chaetotaxy. The head Mac formula differs slightly in *E.strigata*, with more Mac in the H2 and H5 areas. In contrast, there are fewer Mac in the area T1 of Th II and in the area A8 of Abd IV, and there are fewer chaetae also in the manubrial plate (Table 1).

### ﻿ Entomobrya handschini

Taxon classificationAnimaliaEntomobryomorphaEntomobryidae

Stach, 1922

AC6C3F5B-EA52-5990-ABDE-8E0C53141D66

Figs 10 , 11

#### Material.

Five ♂♂ and five ♀♀ on three slides (Nr. HNHM-collpr-919 to HNHM-collpr-920; and WD–coll–149), 17 specimens in 96% ethyl alcohol (Vial WD179). Hungary, Osli, com. Győr-Moson-Sopron, 112 m asl, 47°39'05"N, 17°06'15"E, D-vac sample, 12 Jul. 2020, leg. D. Winkler. Two slides are stored at HNHM, one slide and the specimens in alcohol are preserved at SOE.

#### Description.

**Figure 10.**
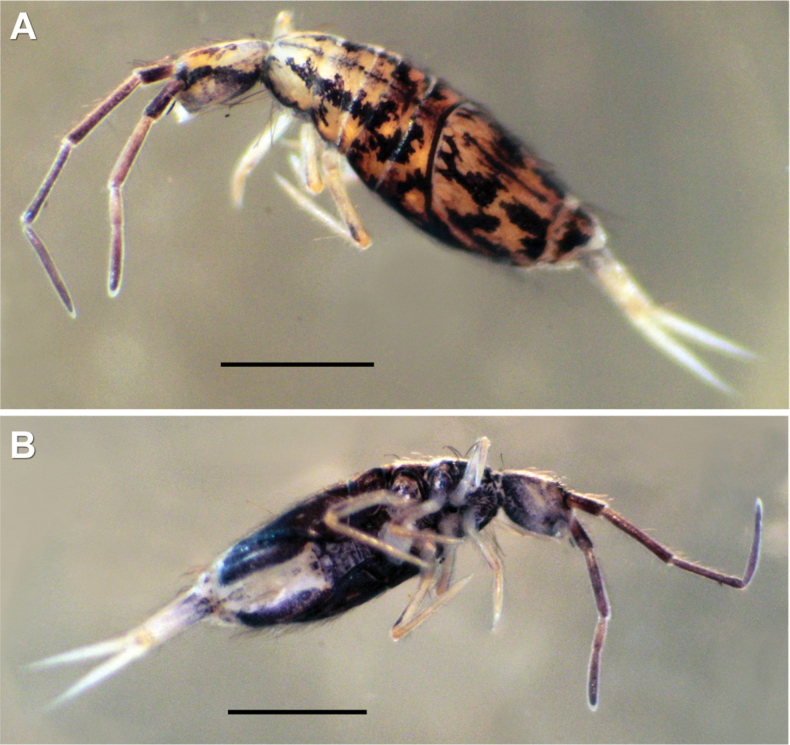
*Entomobryahandschini.* Habitus **A** dorsolateral view **B** ventral view (same specimen). Scale bars: 1 mm.

***Habitus*.** Adult body length up to 3.62 mm excluding antennae. Base colour pale yellow to orange-brown. Colour pattern typically with five longitudinal stripes running from the anterior part of Th II: the central stripe thin and often reaching the posterior part of Abd IV, the dorsolateral stripes reach the mid Abd II. Irregular oblique patches typical for the species on both Abd II and III. Abd IV with four or five irregular patches separated or connected (Fig. 10A). Ventral body entirely dark in most of the adult specimens (Fig. 10B)

**Figure 11.**
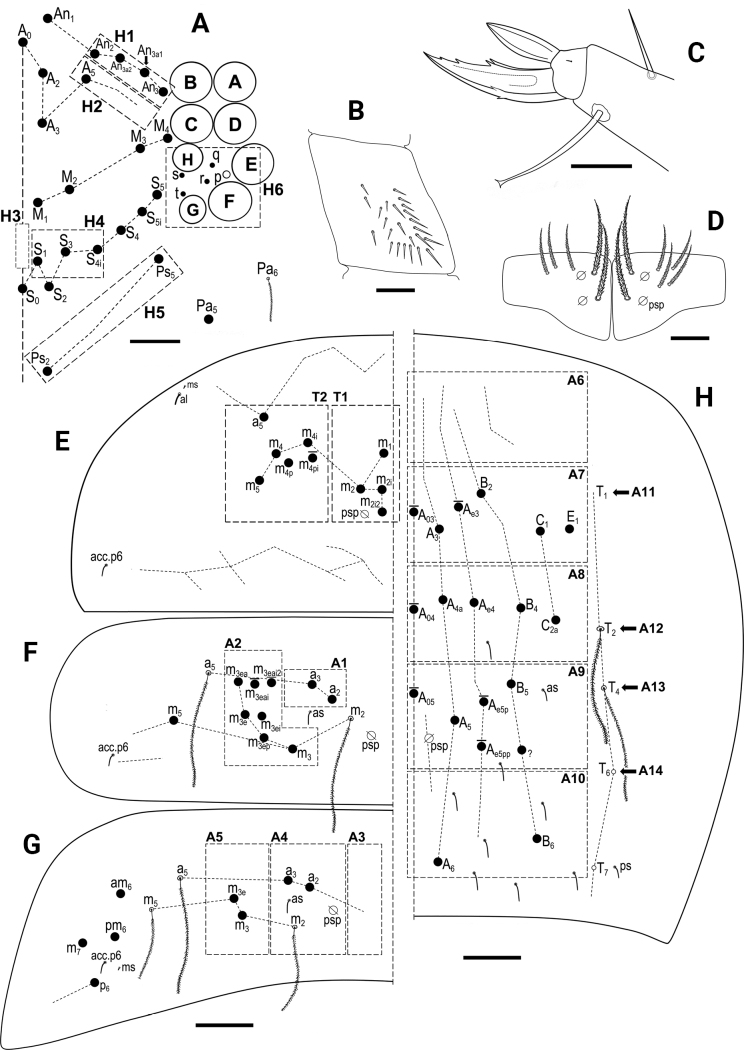
*Entomobryahandschini***A** head chaetotaxy **B** trochanteral organ **C** unguis and unguiculus of leg III **D** manubrial plate **E**Th II dorsal macrochaetotaxy **F**Abd II dorsal macrochaetotaxy **G**Abd III dorsal macrochaetotaxy **H**Abd IV dorsal macrochaetotaxy. Abbreviations: Abd = abdominal tergite; Th = thoracic tergite. Scale bars: 0.03 mm (**A, B, D**); 0.02 mm (**C**); 0.05 mm (**E–H**).

***Head*.** 8+8 eyes, GH smaller than EF (Fig. 11A). Interocular chaetotaxy with five chaetae (s, t, p, q, r). Antennae length 1.30–1.99 mm (*n* = 7). Antennal length to head diagonal length ratio 2.49–3.26 (*n* = 7). Relation of antennal joints I–IV as 1: 2.2: 1.9: 2.2. Ant IV with bilobed apical bulb. Ant III sensillary organ composed of two sensory rods partially behind a cuticular fold, guarded by three short sensilla. Arrangement of chaetae on labrum 4/554, prelabral chaetae ciliated, posterior, median and anterior labral chaetae smooth. Labrum with four labral papillae with spine-like expansion (as in Fig. 8B). Outer maxillary palp with two smooth chaetae and three smooth sublobal chaetae. Lateral process on labial papilla E not reaching apex of papilla (as in Fig. 3E). Labium chaetotaxy formed by 5 smooth “a” chaetae and, in basal row, by ciliated chaetae M, R, E, L_1_, and L_2_ with R smaller than other chaetae (ratio of R/M~0.7).

***Body*.** Ratio of Abd IV/III length 3.14–3.89 (*n* = 9). No differentiated chaetae on tibiotarsus III, except for smooth terminal chaeta opposite to tenent hair. Trochanteral organ with up to 24 spine-like chaetae (Fig. 11B). Unguis with sub-equal paired basal teeth at 52% from the inner edge and with two more unpaired teeth at 76% and 89% from inner edge, respectively. Unpaired dorsal and paired lateral teeth intermediate, at level slightly below the paired internal teeth. A small pretarsal chaeta present on both anterior and posterior surfaces. Unguiculus lanceolate, outer lamella smooth (Fig. 11C). Tibiotarsal tenent hair clavate, as long as claw. Ratio of smooth terminal chaeta / unguiculus 0.9. Ventral tube with 17+17 ciliated chaetae on anterior side and 9+9 ciliated chaetae on posterior side; lateral flap with 6 ciliated and 11 smooth chaetae. Manubrial plate with 5–6 chaetae and two psp (Fig. 11D). Length of not ringed terminal dens ~ 2.5× the length of mucro. Mucro with apical tooth markedly larger than anteapical; basal spine just reaching tip of anteapical tooth.

***Macrochaetotaxy*** (Fig. 11A, E–H). The studied population can be described by the following abbreviated formula: 4-1-0-3-2/4-5(6)/2-5(7)/0-2-2/0-1_0_(0)4(5)-1_0_(0)4-1_0_(0)3(5)-2.

Head (Fig. 11A): H1 area with four Mac (An_2_, An_3a1_, An_3a2_, An_3_); H2 area with one Mac (A_5_); H3 area without Mac; H4 area with three Mac (S_1_, S_3_, S4_i_); H5 area with two Mac (Ps_2_ and Ps_5_). Mesothorax (Fig. 11E): area T1 with four Mac (m_1_, m_2_, m_2i_, m_2i2_); T2 with 5–6 Mac (a_5_, m_4_, m_4i_, m_4p_, m_5_ always present, m_4pi_ present or absent). Abdomen: Abd II (Fig. 11F) area A1 with two Mac (a_2_ and a_3_); area A2 with 5–7 Mac (m_3_, m_3e_, m_3ep_, m_3ei_, m_3ea_ always present, m_3eai_ and m_3eai2_ present or absent); Abd III (Fig. 11G) area A3 without Mac; area A4 with two Mac (a_2_ and a_3_), and area A5 also with two Mac (m_3_ and m_3e_); Abd IV (Fig. 11H) area A6 without Mac; area A7 with unpaired central Mac A_03_ present or absent, and with 4–5 Mac (A_3_, B_2_, C_1_, E_1_ always present; A_e3_ present or absent); area A8 with unpaired central Mac A_04_ present or absent, and with four Mac (A_4a_, A_e4_, B_4_, C_2a_); area A9 with unpaired central Mac A_05_ present or absent, and with 3–5 Mac (A_5_, B_5_, and one Mac of uncertain homology always present, A_e5p_ and A_e5pp_ present or absent); and area A10 with two Mac (A_6_ and B_6_); sensillar formula from Th II to Abd V: 2,2/1,2,2,10,3; microsensillar formula from Th II to Abd III: 1,0/1,0,1.

#### Ecology.

The specimens were collected in a secondary hay meadow with woody patches.

#### Remarks.

Baquero et al. (2008) presented a comprehensive redescription of *E.handschini*, providing a large set of characters (including macrochaetotaxy) from specimens from five different localities (Austria, Crete, Greece, Iran, and Slovakia). Variations in colour pattern are presented in several works, however, neither the original description (Stach 1922) nor the subsequent redescriptions (Stach 1963; Baquero et al. 2008; Jordana 2012) mention the possible occurrence of a dark-coloured ventral side, which is presented in this paper for the first time. Further differences compared to previous descriptions include slight variations in the macrochaetotaxy of some areas. There is one more Mac (a total of four) in the area H1 (head). However, this phenomenon is also observed in other *Entomobrya* species, especially in larger individuals, where the antennal series can include more macrochaetae (Furgoł 2017). T2 area (Th II) can have one more Mac, while A2 area (Abd II) can bear two more additional Mac than previously reported (Baquero et al. 2008; Jordana 2012). On Abd IV, the presence of unpaired macrochaetae has not previously been reported, while in the studied specimens, we occasionally detected unpaired chaetae in areas A7, A8, and A9, respectively. The number of Mac in the A9 area can reach up to five, including a Mac of uncertain homology always present in the B row. The same Mac was also indicated by Baquero et al. (2008) as A_e5pp_ shifted outwards.

The abovementioned differences can be considered as slight variations. Taking them into account, the species *E.handschini* can be characterised by the following simplified formula: 3(4)-1-0-3-2/4-5(6)/2-5(7)/0-2-2/0-1_0_(0)3(5)-1_0_(0)4-1_0_(0)3(5)-2.

### ﻿ Entomobrya cf. quinquelineata

Taxon classificationAnimaliaEntomobryomorphaEntomobryidae

Börner, 1901

E1174CA1-2B51-513D-842D-EE4DB290DF77

Figs 12 , 13

#### Material.

Five ♂♂ on two slides (Nr. HNHM-collpr-922 to Nr. HNHM-coll-923) preserved at HNHM, Hungary, Szárhalmi forest, Sopron, com. Győr-Moson-Sop­ron, 47°41'54"N, 16°38'22"E, 214 m above sea level. D-vac sample, 13 Oct. 2018, leg. D. Winkler.

#### Description.

**Figure 12.**
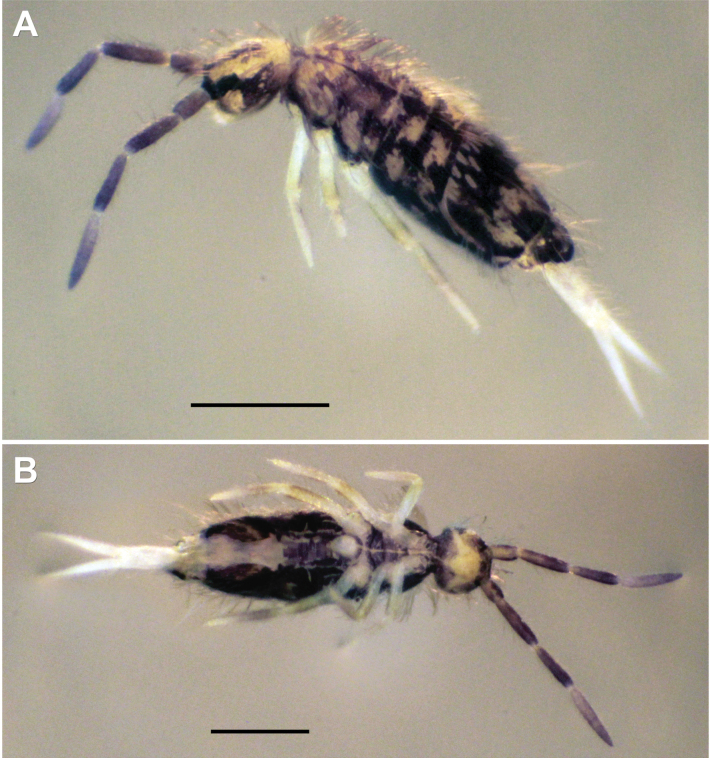
*Entomobrya* cf. quinquelineata. Habitus **A** dorsolateral view **B** ventral view (same specimen). Scale bar: 0.5 mm.

***Habitus*.** Adult body length 1.73–2.05 mm excluding antennae. Body ground colour pale yellow or yellowish brown, with five dark longitudinal stripes: the dorsal and two dorsolateral from Th II to posterior margin of Abd III, lateral ones from Th II to posterior margin of Abd IV. Dorsal and dorsolateral stripes may widen here and there, especially on Th III–Abd III. Abd IV with some irregular patches, anterior part often entirely dark (Fig. 12A). Ventral body partly or entirely dark in most adult specimens (Fig. 12B). Violet pigments on antennae with increasing intensity from base to apex of segments.

**Figure 13.**
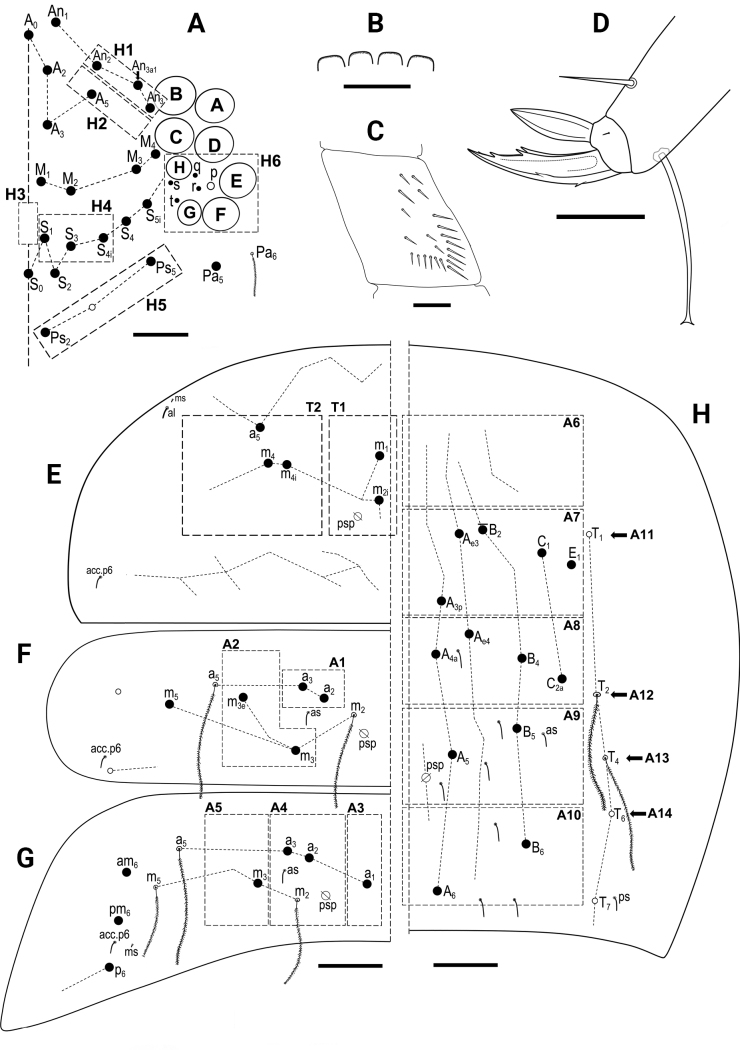
Entomobryacf.quinquelineata**A** head chaetotaxy **B** labral papillae **C** trochanteral organ **D** unguis and unguiculus of leg III **E**Th II dorsal macrochaetotaxy **F**Abd II dorsal macrochaetotaxy **G**Abd III dorsal macrochaetotaxy **H**Abd IV dorsal macrochaetotaxy. Abbreviations: Abd = abdominal tergite; Th = thoracic tergite. Scale bars: 0.03 mm (**A–C**); 0.02 mm (**D**); 0.05 mm (**E–H**).

***Head*.** 8+8 eyes, GH smaller than EF (Fig. 13A). Interocular chaetotaxy with five chaetae (s, t, p, q, r). Antennae length 1.11–1.20 mm. Antennal length to head diagonal length ratio 2.58–2.95 (*n* = 5). Relation of antennal joints I–IV as 1: 2.1–2.3: 1.5–2.0: 1.7–2.5 (*n* = 5). Ant IV with bilobated apical bulb. Ant III sensillary organ composed of two sensory rods partially behind a cuticular fold, guarded by three short sensilla. Arrangement of chaetae on labrum 4/554, prelabral chaetae ciliated, posterior, median and anterior labral chaetae smooth. Labrum with four rounded labral papillae (Fig. 13B). Outer maxillary palp with two smooth chaetae and three smooth sublobal chaetae. Curved lateral process on labial papilla E not reaching apex of papilla. Labium chaetotaxy formed by five smooth “a” chaetae and, in basal row, by ciliated chaetae M, R, E, L_1_, and L_2_ with R smaller than other chaetae (ratio of R/M ~0.6).

***Body*.** Ratio of Abd IV/III length 3.77–4.54 (*n* = 5). Trochanteral organ with up to 22 spine-like chaetae (Fig. 13C). Unguis and unguiculus of claw III as in Fig. 13D. Unguis with sub-equal paired basal teeth at 59% from the inner edge, and with two more unpaired teeth at 80% and 92% from inner edge, respectively. Paired lateral teeth intermediate at level slightly below the paired internal teeth. Unpaired dorsal tooth basal, located approximately at one-quarter of distance from base. A small pretarsal chaeta present on both anterior and posterior surfaces. Unguiculus lanceolate, outer lamella smooth. Tibiotarsal tenent hair clavate, longer than claw. Ratio of smooth terminal chaeta / unguiculus ~ 1. Ventral tube anteriorly with 11+11 chaetae (4+4 ciliated Mac and 7+7 finely ciliated mic) and with 4+4 thin, finely ciliated chaetae on posterior side; lateral flap with 3 ciliated and 6 smooth chaetae. Manubrial plate with four chaetae and two pseudopores. Length of not ringed terminal dens ~ 3× the length of mucro. Mucro with distal tooth equal to anteapical; basal spine just reaching tip of anteapical tooth.

***Macrochaetotaxy*** (Fig. 13A,E–H). Simplified Mac formula: 3-1-0-2(3)-2/2-3/2-2/1-2-1/0-4(5)-4-2-2. Head (Fig. 13A): H1 area with three Mac (An_2_, An_3a1_, An_3_); H2 area with one Mac (A_5_); H3 area without Mac; H4 area with 3 Mac (S_1_, S_3_, S_4i_); H5 area with two Mac (Ps_2_ and Ps_5_). Mesothorax (Fig. 13E): area T1 with two Mac (m_1_ and m_2i_); T2 with three Mac (a_5_, m_4_ and m_4i_). Abdomen: Abd II (Fig. 13F) area A1 with two Mac (a_2_ and a_3_); area A2 also with two Mac (m_3_ and m_3e_); Abd III (Fig. 13G) area A3 with one Mac (a_1_); area A4 with two Mac (a_2_ and a_3_), and one Mac (m_3_) on A5; Abd IV (Fig. 13H) area without Mac; area A7 with 4–5 Mac (A_3p_, A_e3_, C_1_, E_1_ always present, B2 present or absent); area A8 with four Mac (A_4a_, A_e4_, B_4_, C_2a_); area A9 with two Mac (A_5_ and B_5_); and area A10 with two Mac (A_6_ and B_6_). Sensillar formula from Th II to Abd V: 2,2/1,2,2,9,3; microsensillar formula from Th II to Abd III: 1,0/1,0,1.

#### Ecology.

The specimens were collected in xerophilous dolomite-steppe meadow plant associations.

#### Remarks.

The specimens collected in Hungary differ in their colour pattern from the original form described by Börner (1901), which Stach (1963) later named f. principalis, and in which the longitudinal central and dorsolateral stripes run uninterrupted to the posterior part of Abd IV. The similarity in the colour pattern can be discovered mostly with individuals from Switzerland, Holland, or Lithuania, presented in the species redescription (Baquero and Jordana 2008), in which the longitudinal stripes are interrupted on Abd IV. Nevertheless, the occurrence of a dark ventral side has not been described in this species until now, although it is not rare in some populations.

In their comprehensive redescription of *E.quinquelineata* based on European materials, Baquero and Jordana (2008) provided the essential dorsal macrochaetae distribution and information on its variability. The dorsal macrochaetotaxy of the Hungarian specimens is fairly consistent with the previous redescriptions (Baquero and Jordana 2008; Jordana 2012). Differences include the presence of Mac S_1_ in the H4 area of the head. The number of macrochaetae in this area is usually two. However, in a specimen from Switzerland, the presence of a third macrochaeta was hinted at but marked as questionable because of the poor condition of the slide examined (Baquero and Jordana 2008).

Although the dorsal colour pattern suggests that we found a population of *E.quinquelineata*, considering the abovementioned difference in head chaetotaxy, we identify it as E.cf.quinquelineata.

### ﻿ Entomobrya unostrigata

Taxon classificationAnimaliaEntomobryomorphaEntomobryidae

Stach, 1930

BF8EAF45-DE02-5E92-A6EB-EE16636CD03A

Figs 14 , 15

#### Material.

Four ♂♂ and four ♀♀ on three slides (slide numbers as HNHM-collpr-923 to HNHM-collpr-924; and WD–coll–150), ~ 80 specimens in 96% ethyl alcohol (Vial WD180). Hungary, Budapest, 307 m asl, 47°29'14"N, 18°59'23"E, D-vac sample, 14 Mar. 2023, leg. D. Winkler. Two slides are preserved at HNHM, one slide and the specimens in alcohol are stored at SOE.

#### Description.

**Figure 14.**
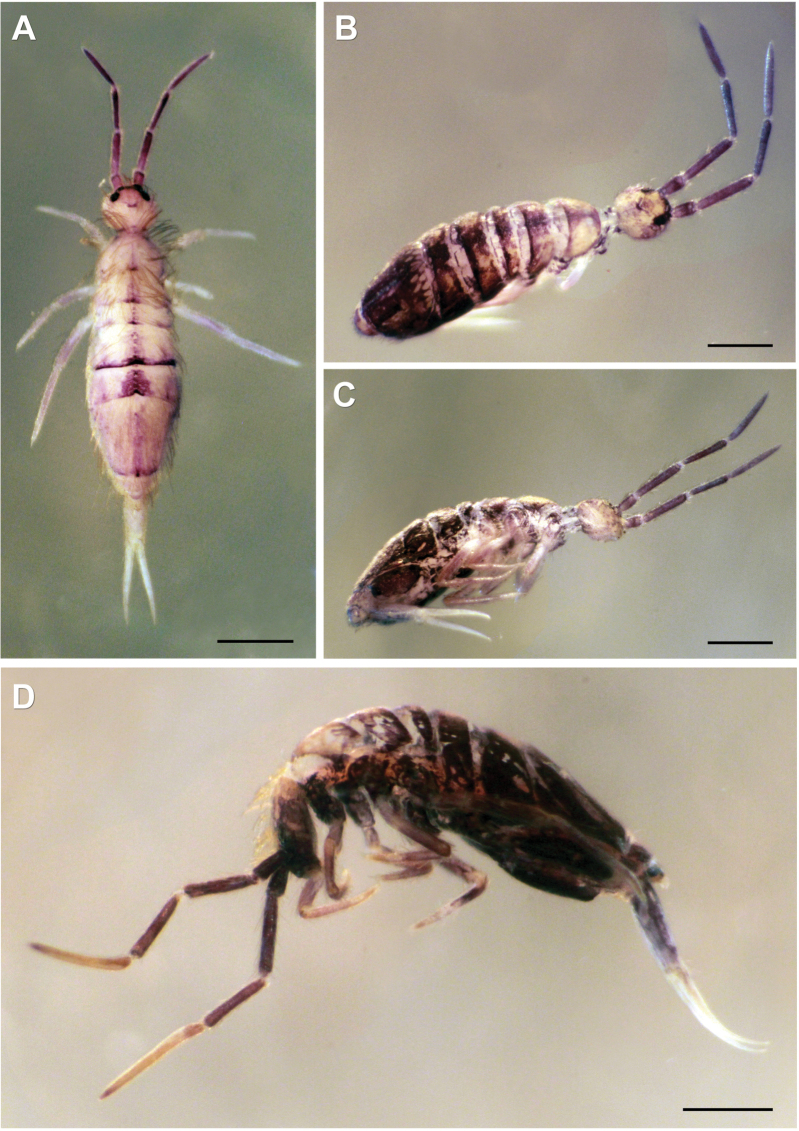
*Entomobryaunostrigata.* Habitus **A** typical colour pattern (dorsal view) **B** intermediate semi-dark colour form (dorsolateral view) **C** intermediate semi-dark colour form (ventrolateral view – same specimen as Fig. 13B) **D** dark colour form (ventrolateral view). Scale bars: 0.5 mm.

***Habitus.*** Adult body length up to 3.91 mm excluding antennae. Colour polymorphic. Ground colour usually pale yellow (Fig. 14A). Transitional form (Fig. 14B, C) with yellow head and Th II, and dark purple from Th III–Abd IV and dark ventral side not rare. Completely dark form (Fig. 14D) occasional, with head and body dorsally and ventrally dark purplish black, dark shades also on ventral tube and manubrium. Pattern typically with narrow medial longitudinal stripe extending from Th II to posterior margin of Abd IV. Broad transverse stripe on posterior margin of Abd II, occasionally also with thin transverse stripes on posterior margins of Th II–Abd I and Abd III–IV. Posteriorly on Th II–Abd III, often with irregular or trapezoidal patches along centreline, usually broadest on Abd III. Pattern on darker specimens barely detectable.

**Figure 15.**
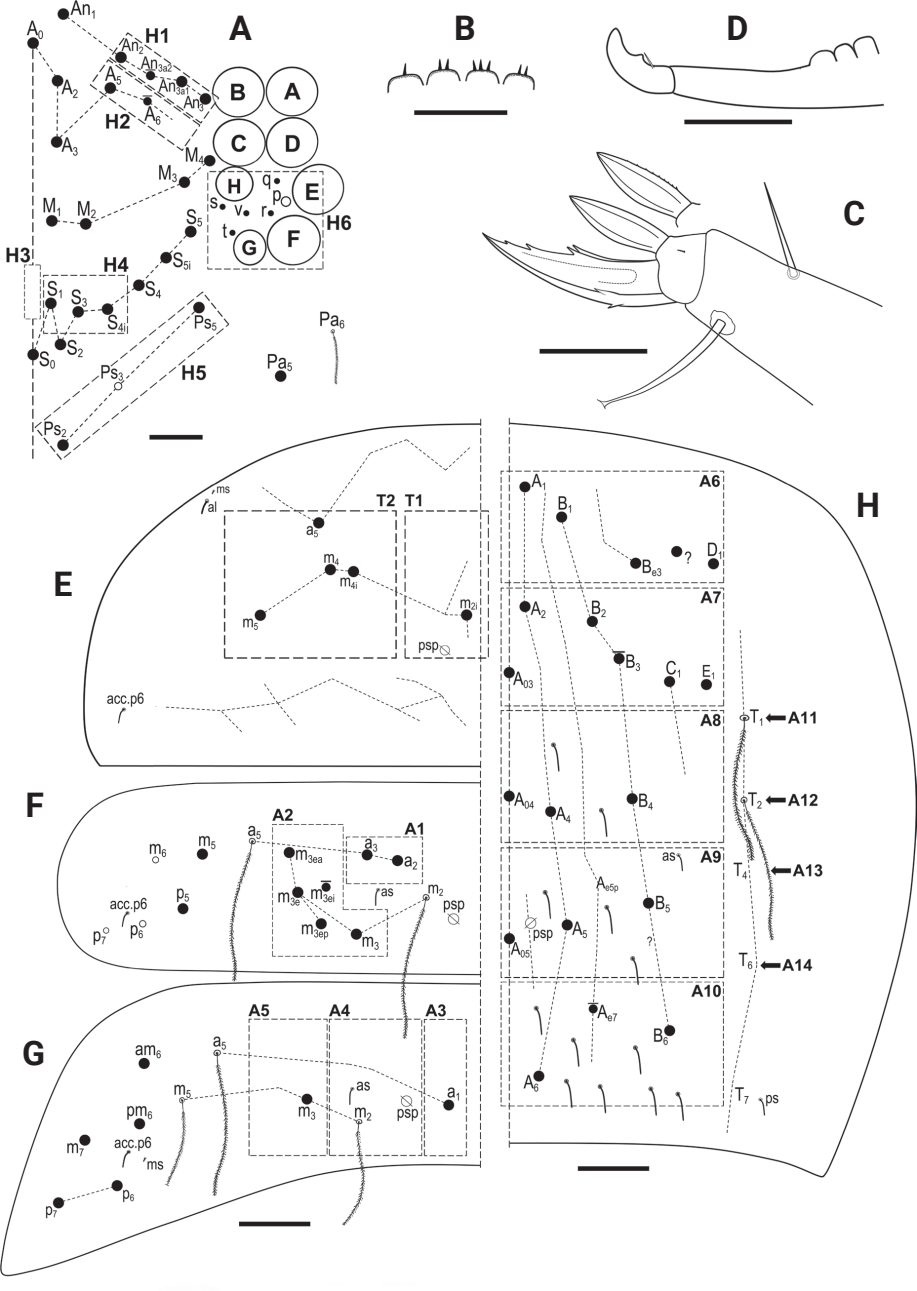
*Entomobryaunostrigata***A** head chaetotaxy **B** labral papillae **C** claw III (unguiculus with two different morphologies: outer edge smooth or serrate) **D** mucro **E**Th II dorsal macrochaetotaxy **F**Abd II dorsal macrochaetotaxy **G**Abd III dorsal macrochaetotaxy **H**Abd IV dorsal macrochaetotaxy. Abbreviations: Abd = abdominal tergite; Th = thoracic tergite. Scale bars: 0.03 mm (**A, C**); 0.02 mm (**B, D**); 0.05 mm (**E–H**).

***Head*.** 8+8 eyes, GH smaller than EF, Interocular chaetotaxy with six chaetae (s, t, p, q, r, v) (Fig. 15A). Antennae length 1.80–2.27 mm (*n* = 7). Antennal length to head diagonal length ratio 2.56–3.20 (*n* = 7). Relation of antennal joints I–IV as 1: 2.1–2.3: 1.8–2.1: 2.4–3.0 (*n* = 7). Ant IV with bilobed apical bulb. Ant III sensillary organ composed of two sensory rods partially behind a cuticular fold, guarded by three short sensilla. Arrangement of chaetae on the labrum 4/554, prelabral chaetae ciliated, posterior, median and anterior labral chaetae smooth. Labrum with four labral papillae with 1–3 setulae expansion (Fig. 15B). Outer maxillary palp with two smooth chaetae and three smooth sublobal chaetae. Lateral process on labial papilla E barely reaching or slightly beyond apex of papilla. Labium chaetotaxy formed by 5 smooth “a” chaetae and, in the basal row, by ciliated chaetae M, R, E, L_1_ and L_2_ with R smaller than other chaetae (ratio of R/M~0.6).

***Body*.** Ratio of Abd IV/III length 4.04–5.57 (*n* = 8). No differentiated chaetae on tibiotarsus III, with exception of the smooth terminal chaeta opposite to tenent hair. Trochanteral organ with up to 33 spine-like chaetae forming a +/– V-shaped pattern. Unguis with sub-equal paired basal teeth at 47% from the inner edge, and with two more unpaired teeth at 74% and 87% from inner edge, respectively. Unpaired dorsal and paired lateral teeth intermediate, at a level below the paired internal teeth. A small pretarsal chaeta present on both anterior and posterior surfaces. Unguiculus lanceolate, outer lamella smooth or serrate (Fig. 15C). Tibiotarsal tenent hair clavate, ~0.8 as long as claw. Ratio of smooth terminal chaeta/unguiculus 0.9. Ventral tube with 30+30 ciliated chaetae on anterior side and 19+19 ciliated chaetae on posterior side; lateral flap with 12 chaetae. Manubrial plate with three chaetae and two psp. Length of not ringed terminal dens ~ 2.5× the length of mucro. Mucro with anteapical tooth markedly smaller than apical; basal spine just reaching tip of anteapical tooth (Fig. 15D).

***Macrochaetotaxy*** (Fig. 15E–H). The studied population can be described by the following abbreviated formula: 3(4)-1(2)-0-3-2/1-4/2-4(5)/1-0-1/4(5)-1_0_4(5)-1_0_2-1_0_2-2.

Head (Fig. 15A): H1 area with 3–4 Mac, An_2_, An_3a1_, and An_3_ always present, An_3a2_ present or absent. H2 area with 1–2 Mac, A_5_ always present, A_6_ present or absent; H3 area without Mac; H4 area with three Mac (S_1_, S_3_, S4_i_); H5 area with two Mac (Ps_2_ and Ps_5_). Mesothorax (Fig. 15E): area T1 with one Mac (m_2i2_); T2 with four Mac (a_5_, m_4_, m_4i_, m_5_). Abdomen: Abd II (Fig. 15F) area A1 with two Mac (a_2_ and a_3_); area A2 with 4–5 Mac (m_3_, m_3e_, m_3ep_, m_3ea_ always present, m_3ei_ present or absent); Abd III (Fig. 15G) area A3 with one Mac (a_1_); area A4 without Mac, and area A5 with one Mac (m_3_); Abd IV (Fig. 15H) area A6 with 4–5 Mac (A_1_, B_1_, B_e3_, D1 always present, one Mac of uncertain homology present or absent; area A7 with unpaired central Mac A_03_, and with 4–5 Mac (A_2_, B_2_, C_1_, E_1_ always present; B_3_ present or absent); area A8 with unpaired central Mac A_04_, and two Mac (A_4_ and B_4_); area A9 with unpaired central Mac A_05_, and two Mac (A_5_ and B_5_); and area A10 with two Mac (A_6_ and B_6_); sensillar formula from Th II to Abd V: 2,2/1,2,2,14,3; microsensillar formula from Th II to Abd III: 1,0/1,0,1.

#### Ecology.

The species was found in an urban park (grass habitat) in considerable abundance.

#### Remarks.

Originally described from the Spanish mainland (Stach 1930), *E.unostrigata* has since been detected mainly from southern European countries, such as Italy (Simon 1965), France (Renaud et al. 2004), and Bulgaria (Tsonev and Kazandzhieva 1991), as well as introduced to North America and Australia (Christiansen 1956; Greenslade 1995). In Hungary, this is the first record of the species. The fact that it was found in the capital, where it proved to be extremely abundant, but not anywhere else, despite the extensive Collembola collections that have taken place in the country in the past decades, raises the idea that the species was also introduced in Hungary.

The specimens collected show great variability in terms of pattern and colour form. In the original (Stach 1930) and later descriptions (Stach 1963), the author distinguished three forms based on the colour pattern. Apart from the typical principal form, a slightly different pattern was described as var. dorsosignata, as well as a pale-coloured form (ab. astrigata) without any pattern but a small dot between the antennae. Further variations in colour pattern and pigmentation have been documented by, e.g., Wray (1953), Christiansen (1958), Simón (1976), Christiansen and Bellinger (1980), Greenslade (1995), and more recently by Katz et al. (2015), Baquero and Jordana (2018), and Jordana and Greenslade (2020). Dark lateral pigmentation on the edges of certain tergites has already been noted (e.g., Stach 1963; Simón 1976; Katz et al. 2015), and darker pigmented specimens have also been presented in some papers (Greenslade 1995). Nevertheless, specimens with dark ventral sides or completely dark specimens with hardly visible patterns (as in Fig. 14C, D) we found in the sampled Hungarian population have not been described yet.

Large individuals are not uncommon in the studied Hungarian material; the maximum length (without antennae and furca) almost reaches 4 mm, while, according to the data published so far, the species is smaller: specimens up to 2 mm from the Spanish mainland (Stach 1963), up to 2.5 mm from the United States (Christiansen and Bellinger 1980), up to 2.62 mm from the Canary Islands (Baquero and Jordana 2018), and up to 2.35 from Australia (Jordana and Greenslade 2020), respectively. Similarly to European and Australian specimens, the eyes G and H are smaller compared to C and F, while they are similar in size in the case of specimens from the USA (Katz et al. 2015).

Slight variations in the macrochaetotaxy of some areas can also be detected compared to previous descriptions (Katz et al. 2015; Baquero and Jordana 2018; Jordana and Greenslade 2020). Larger specimens often have four Mac in the H1 area of the head. In some specimens, there is one more additional Mac also in the H2 area. Similarly to the specimens from North America, we found four or five Mac in area A2 of Abd II, while individuals from Australia and other European regions bear three or four macrochaetae in this area. On Abd IV, the unpaired macrochaetae A_03_–A_05_ are present in the Hungarian individuals (the absence of these macrochaetae has only been documented in North American individuals (Katz et al. 2015)). The number and homology of macrochaetae in the areas A6–A10 roughly correspond to those documented in previous redescriptions (Katz et al. 2015; Baquero and Jordana 2018; Jordana and Greenslade 2020). Considering all these variations, the species *E.unostrigata* can be characterised by the following simplified formula: 3(4)-1(2)-0-3-2(3)/1-4/2-4(3–5)/1-0-1/4(2–5)-1_0_(0)4(5)-1_0_(0)1(2)-1_0_(0)2(1–3)-2(3).

## ﻿Conclusions

In addition to the description of the new species *E.arenaria* sp. nov. and the redescription of *E.nigriventris*, our study aimed to provide an overview of European *Entomobrya* species in which the dark ventral side may occur. So far, this character has only been mentioned in the literature for *E.nigriventris*, which has the potential for misidentification. In addition to the species included in this paper, other *Entomobrya* species also have colour forms in which a dark ventral side is likely to be present, including the dark form of E.multifasciataf.nigra (Stach 1963; Jordana 2012) and the dark from of *E.schoetti*, clearly illustrated by Jordana and Baquero (1999) in the species redescription. Although we have not been able to collect the dark forms of these two species in Hungary, colour forms with dark ventral side have also been found in *E.hanschini*, *E.quinquelineata*, *E.unostrigata*, and the newly described *E.arenaria* sp. nov. Considering the new species and *E.unostrigata*, which is new to the Hungarian fauna, we present an updated list of species of the genus *Entomobrya* (22 species in total) that have been detected in Hungary so far:

1. *Entomobryaalbanica* Stach, 1922

2. *Entomobryaalbocincta* (Templeton, 1835)

3. *Entomobryaarborea* (Tullberg, 1871)

4. *Entomobryaarenaria* sp. nov.

5. *Entomobryaatrocincta* Schött, 1896

6. *Entomobryacorticalis* (Nicolet, 1842)

7. *Entomobryadorsalis* Uzel, 1891

8. *Entomobryahandschini* Stach, 1922

9. *Entomobryalanuginosa* (Nicolet, 1842)

10. *Entomobryamarginata* (Tullberg, 1871)

11. *Entomobryamultifasciata* (Tullberg, 1871)

12. *Entomobryamuscorum* (Nicolet, 1842)

13. *Entomobryanicoleti* (Lubbock, 1868)

14. *Entomobryanigriventris* Stach, 1929

15. *Entomobryanivalis* (Linnaeus, 1758)

16. *Entomobryapazaristei* Denis, 1933

17. *Entomobryaquinquelineata* Börner, 1901

18. *Entomobryaschoetti* Stach, 1922

19. *Entomobryaspectabilis* Reuter, 1890

20. *Entomobryasuperba* (Reuter, 1876)

21. *Entomobryaunostrigata* Stach, 1930

22. *Entomobryaviolaceolineata* Stach, 1963

## Supplementary Material

XML Treatment for Entomobrya arenaria

XML Treatment for Entomobrya nigriventris

XML Treatment for Entomobrya handschini

XML Treatment for Entomobrya cf. quinquelineata

XML Treatment for Entomobrya unostrigata

## ﻿Additional information

### Author ORCIDs

Daniel Winkler https://orcid.org/0000-0002-6008-0562

Jakub Sternalski https://orcid.org/0000-0002-7560-0970

Gábor Ónodi https://orcid.org/0000-0003-2119-4695

Nóra Szigeti https://orcid.org/0000-0003-2483-6761

Norbert Florián https://orcid.org/0000-0001-7585-6709

László Dányi https://orcid.org/0000-0003-0646-2639

### Data availability

All accession numbers and links for the data that support the findings of this study are available in the main text.
